# Determinants of childhood diarrhea in Medebay Zana District, Northwest Tigray, Ethiopia: a community based unmatched case–control study

**DOI:** 10.1186/s12887-018-1098-7

**Published:** 2018-03-29

**Authors:** Kiflom Fisseha Asfaha, Fessahaye Alemseged Tesfamichael, Gizienesh Kahsay Fisseha, Kebede Haile Misgina, Meresa Gebremedhin Weldu, Negassie Berhe Welehaweria, Yosef Sibhatu Gebregiorgis

**Affiliations:** 1 Tigray Regional State, Education Bureau, Mekelle, Ethiopia; 2 Department of Public Health, College of Health Sciences, Aksum University, Aksum, Ethiopia; 3 College of Health Sciences, Jimma University, Jimma, Ethiopia; 4 School of Public Health, College of Health Sciences, Mekelle University, P.O.Box: 1871, Mekelle, Ethiopia

**Keywords:** Diarrhea, Index child, Surveillance, District, Determinants

## Abstract

**Background:**

Globally, childhood diarrhea is amongst the most prevalent health problems and accounts for 9% of all deaths in children under-five. In Ethiopia, childhood diarrhea is a major public health problem in which the prevalence ranges from 13.5 to 30.5% and experienced by multiple factors. Although health extension program has been implementing for couples of years; diarrheal disease remains the second cause of morbidity and continues an important public health problem in the study district. Thus, this study assessed determinants of diarrheal disease among under-five children in the Medebay Zana district, northwest Tigray, Ethiopia, 2015.

**Method:**

A community based case-control study was used. A multistage sampling procedure was employed to enroll the study participants. Data were collected using face to face administered questionnaire. The collected data were entered into Epi Info version 3.5.4 and exported to SPSS Version 21 for analysis. Frequencies with percentages were computed using univariate analysis. Finally, logistic regression model was fitted to identify factors associated with childhood diarrhea where *P*-values of less than 0.05 were considered statistically significant.

**Results:**

Socio-demographic factors such as low maternal educational level [AOR = 2.88, 95% CI (1.70, 4.88)], being index child of older age, households with ≥3 number of children under-five [AOR = 4.05, 95% CI (1.91, 8.60)] and maternal history of diarrhea [AOR = 2.10, 95% CI (1.09, 4.05)] were statistically associated with childhood diarrhea. This study also revealed that child feeding practice; not exclusively breastfed [AOR = 4.84, 95% CI (2.21, 10.60)] and initiation of complementary feeding above 6 months [AOR = 1.78, 95% CI (1.09, 2.92)] were statistically associated with outcome variable. Environmental and behavioral practices such as unavailability toilet facility [AOR = 2.10, 95% CI (1.34, 3.30)], improper solid waste disposal [AOR = 2.29, 95% CI (1.53, 3.44)] and unprotected drinking water [AOR = 1.83, 95% CI (1.12, 2.98)] were also found significant factors of childhood diarrhea.

**Conclusion:**

Maternal educational status, age of index child, number of < 5 children, child feeding practices, maternal history of diarrhea, toilet facility, solid waste disposal and household drinking water were found determinants of childhood diarrhea. These findings have policy implications and insight the strengthening for health intervention programs.

## Background

Diarrhea in children is defined as the occurrence of three or more loose or liquid stools per day by the World Health Organization (WHO). Frequent occurrence of formed stools is not diarrhea, nor is the passing of loose, “pasty” stools by breastfed babies [1]. Diarrheal diseases are amongst the most prevalent health problems in under-five children accounting for 9% of all deaths worldwide in 2015 [2]. The WHO revealed that worldwide, approximately 1.7 billion cases and 760,000 deaths of childhood diarrhea occur each year [1]. Most childhood deaths from diarrhea occur among children less than 2 years of age living in poor settings of sub-Saharan Africa and South Asia [2]. The Southeast Asian and African regions each contributed 26% of severe episodes of diarrhea in 2010 [3]; as a result in the burden of diarrheal diseases in developing countries is greater than in developed countries [3, 4]. According to UNICEF in 2016, the total annual childhood diarrhea deaths reduced by more than 50% (for the last 15 years, decreased from over 1.2 million to half a million) [2]. This is miserable since the problem can be easily treated with oral rehydration therapy (ORS) [3].

In poor settings of sub-Saharan Africa, 25 to 75% childhood illness and 50% childhood deaths occurred due to diarrhea [3]. In poor settings of African region, rotavirus contributed the highest child deaths and is remained the main cause of diarrhea [5]. The magnitude of childhood diarrhea in East Africa was 13–32% [6–9]. Reports and studies on child mortality and morbidity in Ethiopia showed that diarrhea is a major public health problem [10, 11]. The 2011 Ethiopian Demographic and Health Survey (EDHS) revealed that 13% of the Ethiopian children had diarrhea in the 2 weeks preceding the survey [12, 13]. Other studies in different parts of Ethiopia indicated that the prevalence of childhood diarrhea was in the range of 13.5–30.5% [14–22].

Multiple factors contribute to the occurrence of diarrhea among children under-five years of age. Childhood diarrhea was significantly associated with maternal related factors: low maternal education [7, 17, 18, 20, 22, 23], mother’s age [9], history of maternal diarrheal morbidity [21], poor knowledge of mothers on diarrhea [19] and rural residence [22], Child related factors such as sex of child [22], age of children [7, 16, 18–20, 23] and malnutrition [21] were statistically associated with childhood diarrhea.

Several studies reported that environmental conditions and behavioral practices; number of under-five children [18, 21], latrine availability [18, 24], supplementary feeding commencing time [19, 23], mode of feeding [21], improper child stool disposing methods [18], mothers not practicing hand washing at important times [17, 19, 20, 22], lack of safe water sources [22, 24, 25], improper handling of water for drinking [15, 23, 25], improper solid waste disposal methods [16, 17], lowest wealth status [19], and longer time elapsed to visit households by health extension workers [19] were also significantly associated with childhood diarrhea. A systematic study conducted in Low and Middle Income Countries (LMICs) also revealed that diarrheal diseases are prevalent in areas with water scarcity, unsafe drinking water supply, poor hygiene, and lack of sanitation [26].

According to EDHS 2011 report, under-five mortality in Tigray region was 85 per 1000 live births [6]. A study conducted in Laelay Michew ditrict of Tigray regional state indicated the prevalence of diarrhea in the community was 17.7% [22]. Despite the high prevalence of diarrhea and higher child mortality in the region, there is limited information about the determinants of childhood diarrhea. According to the study’s district health office report in 2015, diarrheal disease remains the second cause of morbidity and it continues an important public health problem despite the comprehensive health extension programs in place. Identifying the determinants of diarrhea is very important for effective implementation of child health intervention programs, for general assessment of resource requirements and intervention prioritizations. Therefore, this study has assessed the determinants of diarrhea among under-five children in Medebay Zana district, northwest Tigray, Ethiopia.

## Methods

### Study design, setting and population

A community-based unmatched case-control study was employed from October 1 to November 15/2015 among under-five children living in Medebay Zana district. Medebay Zana district is located in northwest of Tigray, Ethiopia at a distance of 272 kms from Mekelle and 1010 kms away from Addis Ababa. Administratively the district is divided in to 20 kebeles (small local administrative units). According to the Central Statistical Agency of Ethiopia 2007 report, the total population of the district is 153,521 with 35,309 reproductive age women (15–49 years of age) and 22,224 under-five children [27]. According to the district’s health office report in 2015; more than half of the residents have no access to improved water supply [28]. The district has one primary hospital, 5 health centers, and 21 health posts. The source population of the study was all under-five children living in Medebay Zana district. Cases and controls were children in the age group of 0–59 months with and without diarrhea in the preceding of 2 weeks during a house to house survey, respectively.

### Sample size and sampling procedures

Sample size was determined using double population proportion formula. The required sample size was calculated using Epi Info software version 6 with the following assumptions: the prevalence of childhood diarrhea derived from a study done by Girma R. et al. in Nekemte town, Ethiopia [29] with 61.47 and 42.86% proportion of controls and cases respectively with exposure (disposal of refuse in the pit), a ratio of case to control of 1:2, 95% confidence level, 5% margin of error and 80% power; generated a sample size of 273. The final sample size required to this study with 2 design effect and 10% contingency was 600 (200 cases and 400 controls).

Multi-stage sampling technique was used; where first six kebeles (small local administrative units) out of 20 were selected using simple random sampling; secondly a house-to-house survey was conducted in the selected kebeles to identify a total of 250 cases and 1513 controls that fulfilled the inclusion criteria and to construct sampling frame prior the actual study. Then, the total sample size was proportionately allocated; cases and controls were selected by simple random sampling method using computer randomizer table.

### Operational definitions

#### Diarrhea

Having three or more loose or watery stools in a 24 hour period, as reported by the mother/caretaker of the child.

#### Soap use for hand washing at critical time

If a mother/caregiver practiced either simple hand washings with soap either before food preparation, before child feeding, after child cleaning and after latrine visiting was considered as “Yes means all practiced” unless considered as “No partially practiced”.

#### Solid waste disposal methods

Is a way of disposal refuses which includes burning, buried in pit or store in a container, compost, and disposed in designed site was considered as “proper”, whereas disposing in open field was considered as “improper” refuse disposal.

#### Relative wealth to others

A self report on economic status; which includes emergency resources and hunger in the last 1 month; when the respondents reported “No” for emergency resources and “Yes” for hungry was considered as “lower relative wealth to others” otherwise higher relative wealth.

#### Index child

A child that is included in the study from a household to have information on the demographic and health characteristics, and also to calculate the prevalence and incidence of diarrhea.

### Data collection tool and procedure

A pre-tested structured questionnaire was used to interview the mother or caretaker of the index child. The questionnaire tool was constructed after conducting extensive similar literature review [23, 29]. The tool was contextualized and included: socio-demographic, maternal/caregiver and child characteristics, caring practices, environmental conditions and behavioral practices. Data were collected by eight 10th grade data collectors under the supervision of two health professionals. Comprehensive training was given for 2 days. Both data collectors and supervisors did not participate in the survey; were unaware of diarrheal disease status of the study groups and were provided only the identification numbers of households and name and age of the child. The collected data were checked by the principal investigator on a daily basis for any incompleteness and/or inconsistency.

### Data processing and analysis

Data were entered into Epi Info version 3.5.4, and transferred to SPSS version 21 for analysis. The outcome variable was childhood diarrhea which was dichotomized by assigning ‘1’ for those who had diarrhea and ‘2’ for those who had not diarrhea. Descriptive statistics such as frequencies with percentages were computed. Binary logistic regression was modeled; in the bivariable analysis, candidate independent variables for multivariable analysis were selected at *P*-value of less than 0.2. Adjusted Odds Ratios (AOR) with 95% confidence intervals was estimated to assess the strength of the association and a *p*-value less than 0.05 was used to declare the statistical significance in the multivariable analysis. The Hosmer and Lemeshow goodness-of-fit (*p*-value = 0.35) was checked to test for model fitness. The independent variables were tested for multicollineanrity using the Variance Inflation Factor (VIF) and the Tolerance tests. When collinearity occurred; the study considered which variables were worth in terms of biological and literature evidences.

## Results

### Socio-demographic characteristics

A total of 1763 under-five children (250 with diarrhea and 1513 without diarrhea) were identified during a house to house survey. Of these, 600 under five children (200 cases and 400 controls) with their respective mothers or caregivers were sampled for this study with a response rate of 99.5% in both study groups. In the current study more than 95% of the index children, 190 (95.5%) cases and 392 (98.5%) controls were from their real mothers. More than half of the mothers/caregivers in both groups; 106 (53.3%) cases and 217 (54.5%) controls were in the age group of 25–34 years.

Regarding head of households, 184 (92.5%) cases and 379 (95.2%) controls were headed by males. Majority of the children’s mothers/caregivers in both study groups; 173 (86.9%) cases and 267 (67.1%) controls had low educational level. Majority of mothers/caregivers in both groups, 177 (88.9%) cases and 346 (86.9%) controls were housewives or farmers by occupation. Mothers/caregivers were interviewed for their perceived economic status; nearly 90% in both groups; 185 (93.0%) cases and 358 (89.9%) controls reported that they had lower relative wealth to others.

Among these studied children, 70 (35.2%) cases and 109 (27.4%) controls were found in the age group of 12–23 and 91 (45.7%) cases and 157 (39.4%) controls were in the age group of 24 and above months (Table 1).

**Table 1:** Socio-demographic characteristics of respondents in Medebay Zana District, Northwest Tigray, Ethiopia, 2015 (*n* = 597; cases: 199 and controls: 398)

Characteristics	Diarrheal disease status	
	Cases (%)	Controls (%)
Maternal relation with child		
Caregiver	9 (4.5)	6 (1.5)
Mother	190 (95.5)	392 (98.5)
Maternal/Caregiver age		
15–24	29 (14.6)	64 (16.1)
25–34	106 (53.3)	217 (54.5)
35 and above	64 (32.2)	117 (29.4)
Head of household		
Female	15 (7.5)	19 (4.8)
Male	184 (92.5)	379 (95.2)
Maternal educational status (*n* = 582)		
Low educational level	185 (95.5)	343 (88.4)
High educational level	9 (4.5)	45 (11.6)
Maternal/Caregiver occupation		
House wife or Farmer	177 (88.9)	346 (86.9)
Self or paid employee	22 (11.1)	52 (13.1)
Relative wealth to others		
Lower relative wealth	185 (93.0)	358 (89.9)
Higher relative wealth	14 (7.0)	40 (10.1)
Gender of the child		
Male	100 (50.3)	195 (49.0)
Female	99 (49.7)	203 (51.0)
Age of the child in month		
< 6	5 (2.5)	56 (14.1)
6–11	33 (16.6)	76 (19.1)
12–23	70 (35.2)	109 (27.4)
24 and above	91 (45.7)	157 (39.4)
No of under five in the household		
2 or less	173 (86.9)	383 (96.2)
3+	26 (13.1)	15 (3.8)
Child birth order		
1	48 (24.1)	99 (24.9)
2–3	101 (50.8)	176 (44.2)
4–5	24 (12.1)	82 (20.6)
6+	26 (13.1)	41 (10.3)

### Maternal/caregiver and child characteristics and caring practices

Concerning place of delivery of the index child, more than a one third, 93 (46.7%) cases and 126 (31.7%) controls were delivered at home. Less than 5% of the studied children; 6 (3.0%) cases and 19 (4.8%) controls were never been immunized. Regarding feeding practice; 27 (13.6%) cases and 18 (4.5%) controls for exclusive breast and 72 (36.2%) cases and 105 (26.4%) controls for complementary feeding did not follow the recommend practices. This study assessed about maternal/caregiver history of diarrheal disease in the previous 2 weeks preceding the survey; 29 (14.6%) cases and 31 (7.8%) controls had diarrheal disease (Table 2).

**Table 2:** Maternal and child characteristics and caring practices of the respondents in Medebay Zana District, Northwest Tigray, Ethiopia, 2015 (*n* = 597; cases: 199 and controls: 398)

Characteristics	Diarrheal disease status	
	Cases (%)	Controls (%)
Place of delivery		
Home	93 (46.7)	126 (31.7)
Health facility	106 (53.3)	272 (68.3)
Immunization status		
Never been immunized	6 (3.0)	19 (4.8)
Immunized	193 (97.0)	379 (95.2)
Duration the child exclusively breast fed		
Less or greater than 6 months	27 (13.6)	18 (4.5)
= 6 months	172 (86.4)	380 (95.5
Time the child initiated complementary feeding		
Less or greater than 6 months	72 (36.2)	105 (26.4)
Exactly at 6 months	127 (63.8)	293 (73.6)
Maternal diarrhea in the last two weeks		
Yes	29 (14.6)	31 (7.8)
No	170 (85.4)	367 (92.2)

### Environmental conditions and behavioral practices

A significant number of households in the study area; 139 (69.8%) cases and 186 (46.7%) controls have not had toilet facilities. Among the total households interviewed in this study, 122 (61.3%) cases’ households and 151 (37.9%) controls’ households had improper domestic solid waste disposal methods.

Safe and adequate water supply were assessed using household drinking water and distance to bring water; 122 (61.3%) cases and 151 (37.9%) controls used unprotected drinking water; similarly more than one third 74 (37.2%) cases and 171 (43.0%) controls have travelled a round trip distance of more than 30 min to fetch water. Majority of both groups; 168 (84.4%) cases and 295 (74.1%) controls did not use soap for hand washing at critical time. Concerning children’s stool disposal practice; 139 (69.8%) cases and 184 (46.2%) controls were not safe (Table 3).

**Table 3:** Environmental health conditions and behavioral practices of respondents in Medebay Zana District, Northwest Tigray, Ethiopia, 2015 (*n* = 597; cases: 199 and controls: 398)

Characteristics	Diarrheal disease status	
	Cases (%)	Controls (%)
Toilet facility in the household		
No facility	139 (69.8)	186 (46.7)
Traditional or improved toilet	60 (30.2)	212 (53.3)
Refuse disposal method		
Improper	122 (61.3)	151 (37.9)
Proper	77 (38.7)	247 (62.1)
Type of floor of house made from		
Mud	196 (98.5)	385 (96.7)
Cement	3 (1.5)	13 (3.3)
Type of roof of house made from		
Thatched	25 (12.6)	41 (10.3)
Corrugated iron sheet	174 (87.4)	357 (89.7)
Main source of water		
Unprotected	67 (33.7)	70 (17.6)
Protected	132 (66.3)	328 (82.4)
Round trip distance to fetch water		
> 30 min	74 (37.2)	171 (43.0)
= < 30 min	125 (62.8)	227 (57.0)
Soap use for hand washing at critical time		
Yes	31 (15.6)	103 (25.9)
No	168 (84.4)	295 (74.1)
Method of child stool disposal		
Not safe	139 (69.8)	184 (46.2)
Safe	60 (30.2)	214 (53.8)

### Determinants of childhood diarrhea

Finally, maternal educational status, age of index child, number of under-five children, exclusive breastfeeding, complementary feeding initiation time, maternal diarrhea in the last 2 weeks, type of toilet facility, domestic waste disposal methods and household drinking water were independent predictors of childhood diarrhea.

In the multivariable analysis, maternal educational status was associated with childhood diarrhea; children whose mothers had low educational level were three times more likely to develop diarrhea than children whose mothers had high educational level [AOR = 2.88, 95% CI (1.70, 4.88)]. The study revealed that the children’s exposure to diarrhea was increased when they completed their first 6 months of life and the risk was highest at the age of 12–23 months. Those children whose group of ages in months; 6–11 [AOR = 7.48, 95% CI (2.40, 23.32)], 12–23 [AOR = 11.64, 95% CI (3.86, 35.12)] and 24 and above [AOR = 8.97, 95% CI (3.01, 26.68)] were 7–12 times higher risk of developing diarrhea compared to those whose age were less than 6 months. When number of under-five children in the households increased, the risk of diarrhea also increased; children living in households who had three and above under five children were four folds more likely to experience diarrheal disease [AOR = 4.05 95% CI (1.91, 8.60)] compared to children living in households with two or less under five children.

Children who did not exclusively breastfeed experienced diarrheal disease at five times [AOR = 4.84, 95% CI (2.21, 10.60)] more likely compared to those who exclusively breast fed. This study also showed that children who initiated complementary feeding above 6 months of age were 1.78 times more likely to develop diarrhea [AOR = 1.78, 95% CI (1.09, 2.92)] compared to their counterpart. The likelihood of childhood diarrhea among children from mothers/caregivers who had diarrhea in last 2 weeks was increased by two folds [AOR = 2.10, 95% CI (1.09, 4.05)] compared to their counterpart.

Availability of toilet facility in the households was significantly associated with childhood diarrhea. Children from households who had no toilet facility had two times higher risk of having diarrhea than children from household who had traditional or improved toilet facility [AOR = 2.10, 95% CI (1.34, 3.30)]. Among the behavioral practices; domestic waste disposal methods was associated with childhood diarrhea. Children whose mothers/caregivers practiced improper domestic waste disposal methods were 2.29 times more likely to develop diarrhea than children whose mothers/caregivers practiced proper waste disposal [AOR = 2.29, 95% CI (1.53, 3.44)].

Children whose households consumed unprotected drinking water were two times more likely develop diarrhea [AOR = 1.83, 95% CI (91.12, 2.98)] compared to children whose households consumed protected water (Table 4).

**Table 4:** Bivariable and multivariable logistic regression analysis of factors associated with childhood diarrhea in Medebay Zana District, Northwest Tigray, Ethiopia, 2015 (*n* = 597; cases: 199 and controls: 398)

Variables	Diarrheal disease status		COR (95% CI)	AOR (95% CI)
	Cases, *N* (%)	Controls, *N* (%)		
Maternal relation with child				
Caregiver	9 (4.5)	6 (1.5)	3.09 (1.07, 8.82)*	–
Mother	190 (95.5)	392 (98.5)	1	1
Head of household				
Female	15 (7.5)	19 (4.8)	1.63 (0.81, 3.27)^+^	–
Male	184 (92.5)	379 (95.2)	1	1
Maternal educational status (*n* = 582)				
Low educational level	185 (95.5)	342 (88.4)	3.27 (2.06, 5.18)***	2.88 (1.70, 4.88)***
High educational level	9 (4.5)	45 (11.6)	1	1
Age of the child in month				
< 6	5 (2.5)	56 (14.1)	1	1
6–11	33 (16.6)	76 (19.1)	4.86 (1.77, 13.25)*	7.48 (2.40, 23.32)*
12–23	70 (35.2)	109 (27.4)	7.19 (2.75, 18.84)***	11.64 (3.86, 35.12)***
24 and above	91 (45.7)	157 (39.4)	6.49 (2.51, 16.79)***	8.97 (3.01, 26.68)***
No of under five children in household				
2 or less	173 (86.9)	383 (96.2)	1	1
3+	26 (13.1)	15 (3.8)	3.84 (1.98, 7.43)***	4.05 (1.91, 8.60)***
Child birth order				
1	48 (24.1)	99 (24.9)	1	1
2–3	101 (50.8)	176 (44.2)	1.18 (0.78, 1.81)	–
4–5	24 (12.1)	82 (20.6)	0.60 (.34, 1.07)^+^	–
6+	26 (13.1)	41 (10.3)	1.31 (0.72, 2.38)	–
Place of delivery				
Home	93 (46.7)	126 (31.7)	1.89 (1.34, 2.69)***	–
Health facility	106 (53.3)	272 (68.3)	1	1
Duration the child exclusively breast fed				
Not exclusive	27 (13.6)	18 (4.5)	3.31 (1.78, 6.18)***	4.84 (2.21, 10.60)***
Up to 6 months	172 (86.4)	380 (95.5	1	1
Time initiated complementary feeding				
Initiated above 6 months of age	72 (36.2)	105 (26.4)	1.58 (1.09, 2.28)*	1.78 (1.09, 2.92)*
Initiated at 6 months of age	127 (63.8)	293 (73.6)	1	1
Maternal diarrhea in the last two weeks				
Yes	29 (14.6)	31 (7.8)	2.02 (1.18, 3.46)*	2.10 (1.09, 4.05)*
No	170 (85.4)	367 (92.2)	1	1
Toilet facility in the household				
No facility	139 (69.8)	186 (46.7)	2.64 (1.84, 3.79)***	2.10 (1.34, 3.30)***
Traditional or improved toilet	60 (30.2)	212 (53.3)	1	1
Domestic solid waste disposal methods				
Improper	122 (61.3)	151 (37.9)	2.59 (1.83, 3.68)***	2.29 (1.53, 3.44)***
Proper	77 (38.7)	247 (62.1)	1	1
Household drinking water				
Unprotected	67 (33.7)	70 (17.6)	2.38 (1.61, 3.52)***	1.83 (1.12, 2.98)*
Protected	132 (66.3)	328 (82.4)	1	1
Round trip distance to fetch water				
> 30 min	74 (37.2)	171 (43.0)	0.79 (0.55, 1.11)^+^	–
= < 30 min	125 (62.8)	227 (57.0)	1	1
Soap use for hand washing at critical time				
Yes	31 (15.6)	103 (25.9)	1	1
No	168 (84.4)	295 (74.1)	1.89 (1.21, 2.95)**	–

Statistically significant at ^+^ *P* < 0.2 *P < 0.05, **P < 0.01, ***P < 0.001, CI confidence interval, COR crude odds ratio, AOR adjusted odds ratio. Case = under five children with diarrhea Control = under five children without diarrhea

## Discussion

This study has revealed the determinants of diarrhea among under-five children. Maternal educational status, age of index child, number of under-five children in households, exclusive breastfeeding, complementary feeding, maternal/caregiver diarrhea, availability of toilet facilities in households, domestic solid waste disposal methods and household drinking water were found to be statistically associated with childhood diarrhea. This study indicated that children whose mothers had low educational level were three times more likely to concede diarrhea compared to children whose mothers had higher educational level. This finding is consistent with several studies conducted across the world; in Ethiopia [14, 17, 18, 20, 30], in Uganda [7], Ghana [31] and Salvador, north-eastern Brazil [32]. This could be explained by maternal educational status persuades the personal and environmental hygienic practices, child feeding and caring practices, improving living conditions and reducing resistance for new ways of delivery for prevention and control of communicable diseases interventions like health extension programs.

Concerning the age of index children; older age children were more likely to develop childhood diarrhea compared to younger children, 0–5 months of age. Similar findings have been reported from Ethiopia [14, 16, 19, 20, 23, 30], Ghana [33], Tanzania [34], Egypt [35], Uganda [7]. In this study, the very young children 0–5 months particularly those who exclusively breastfed; more than 85% of cases and controls children were exclusively breastfeed and these children are usually safe and protected from exposure of contaminated agents, but the other age groups (6–11, 12–23 and 24 and above months of age) are exposed to different sources of infections because this is a period of excessive motilities while crawling or walking, tendency of placing in any objects to their mouths and particularly in weaning time; children are prone to diarrhea during consuming foods that are prepared unsanitary conditions.

In present study, children from households with more than three under-five children were four times at risk of diarrhea compared to two or less under-five children in the households. This result is in line with other findings done in Ethiopia [16, 18, 21] and Pakistan [36]. This is because when there are higher number of under-five children in the households; mothers/caregivers are influenced to provide adequate and timely childcare; similar evidence was documented in Dakahlia, Egypt that indicated this might be due to the inability of mother/caregiver to care for a large number of children [35].

Regarding maternal/caregiver health and caring practices; child feeding practices were significantly associated with childhood diarrhea in which children who did not exclusively breastfeed and initiated complementary feeding above 6 months of age were five and two-folds at risk of diarrhea, respectively compared to their counterparts. These findings are in agreement with similar findings done elsewhere; duration of breast feeding [14] in Mecha District, Ethiopia; exclusive breastfeeding protects hospitalization due to diarrhea. This study estimated that 53% of diarrhea hospitalizations occurred each month could have been prevented if all infants were exclusively breastfed [37]. A study conducted in Amhara regional state, Ethiopia also revealed that complementary feeding initiated at inappropriate time increased the odds of childhood diarrhea [19] and similar findings were reported from Kotebe area, Addis Ababa, Ethiopia [23] and rural Zimbabwe [38]. This is because breastfeeding and initiation of complementary feeding on 6th months provide protective factors that could help reduce various infections including diarrhea. They are also strengthening the immunity of children which indirectly reduces diarrhea causative organism(s) accidently introduced into supplementary foods during feeding practices and due to unhygienic procedures in the preparation of feeds, materials and types of water used.

This study revealed that maternal/caregiver history of diarrheal illness was significantly associated with childhood diarrhea. Children whose mothers/caregivers had diarrhea in the 2 weeks period preceding the survey were two times more likely to develop diarrhea than children whose mothers/caregivers had no diarrhea in the given period of time. Similar findings have been documented in Ethiopia [14, 21, 39]. This is due mothers or care givers with diarrhea were considered as main source of childhood diarrhea in this case; they are also responsible in food preparation for their family and the immediate providers of childcare. Moreover, this could be due to the care of the child may be compromised if the mother herself is sick.

Out of the environmental and behavioral conditions considered in this study; availability of toilet facility, domestic solid waste disposal methods and household drinking water were significant risk factors for childhood diarrhea. In this study, children from households which had no toilet facilities were two times higher at risk of diarrhea than those from households with traditional or improved toilet.

The same results are found in developing countries; where in Ethiopia latrine facility availability as a community-level factor [14, 19], not owned latrine in the case-control studies of derashe district and Wolaita Soddo town, Southern Ethiopia [40, 41], and having a latrine within the compound in Tanzania [42].

Households with latrine availabilities might be able to dispose human excretal safely and reduces the frequency of contacts of insects with feces. Latrine reduces fecal contamination of the household compound and community environment; as a result reduces susceptibility of family members to diarrheal morbidity.

Concerning domestic solid waste disposal methods, in the current study, children from those families who used improper domestic solid waste disposal were at 2.29 times risk in developing diarrhea compared to their counterparts. This is in line with similar studies conducted in Ethiopia [16, 29] and in Salvador, north-eastern Brazil where garbage dump nearby [32]. The possible explanation could be an improper disposal of refuse serves as a good source for breeding site of insects, in turn may bring pathogens from the refuse site to water and food.

In present study, children who had unprotected drinking water in their households were almost two folds at risk of concede diarrhea compared to those who had protected drinking water. This result is consistent with several studies’ findings done in Ethiopia [40, 41], a case–control study conducted in Pakistan shown that water and sanitation extension program intervention decreased the odds of diarrhea in children [43]. This is because unsafe water supply usually considers as a main source of water borne diseases including diarrhea.

## Conclusion

This study has identified maternal educational status, age of index child, the number of under-five children in households, exclusive breast and complementary feeding practices, maternal/caregiver diarrhea, availability of toilet facility in households, domestic solid waste disposal methods, and household drinking water were found to be determinants for childhood diarrhea. Therefore, the findings have important policy implications for health intervention programs, insight the strengthening of health extension programs in terms of provision of better sanitation and hygiene practices, intensifying family planning services, and effective educational programs to improve individuals’ living standard conditions.

## Abbreviations

AOR: Adjusted Odds Ratio
CI: Confidence Interval
COR: Crude Odds Ratio
EDHS: Ethiopian Demographic Health Survey
ORS: Oral Rehydration Salt
UNICEF: United Nations Children’s Fund
VIF: Variance Inflation Factor
WHO: World Health Organization
